# SD-IDD: Selective Distillation for Incremental Defect Detection

**DOI:** 10.3390/s26051413

**Published:** 2026-02-24

**Authors:** Jing Li, Chenggang Dai, Xiaobin Wang, Chengjun Chen

**Affiliations:** School of Mechanical and Automotive Engineering, Qingdao University of Technology, Qingdao 266000, China; lijing_wuyu@163.com (J.L.); dcg_sdu@163.com (C.D.); xiaobinwang650@gmail.com (X.W.)

**Keywords:** incremental learning, catastrophic forgetting, selective distillation

## Abstract

Surface defects in industrial production are complex and diverse. Therefore, deep learning-based defect detection models must consistently adapt to newly emerging defect categories. The trained models generally suffer from catastrophic forgetting as they learn new defect categories. To address this issue, we propose a selective distillation for incremental defect detection (SD-IDD) model based on GFLv1. Specifically, three selective distillation strategies are proposed, including high-confidence classification distillation, dual-stage cascaded regression distillation, and Intersection over Union (IoU)-driven difficulty-aware feature distillation. The high-confidence classification distillation aims to preserve critical discriminative knowledge of old categories within semantic confusion regions of the classification head, reducing interference from low-value regions. Dual-stage cascaded regression distillation focuses on high-quality anchors through geometric prior coarse filtering and statistical fine filtering, utilizing IoU-weighted KL divergence distillation loss to accurately transfer localization knowledge. IoU-driven difficulty-aware feature distillation adaptively allocates distillation resources, prioritizing features of high-difficulty targets. These selective distillation strategies significantly mitigate catastrophic forgetting while enhancing the detection accuracy of new classes, without requiring access to old training samples. Experimental results demonstrate that SD-IDD achieves superior performance, with mAP_old of 58.2% and 99.3%, mAP_new of 69.0% and 97.3%, and mAP_all of 63.6% and 98.3% on the NEU-DET and DeepPCB datasets, respectively, surpassing existing incremental detection methods.

## 1. Introduction

Compared with traditional defect detection methods, deep learning-based approaches achieve higher accuracy and better robustness, and have been widely applied in industrial defect detection [1,2,3]. However, they require comprehensive datasets covering all defect categories for training [4]. In real-world production environments, new defect classes continually emerge due to process changes, raw material replacement, and other factors [5]. The defect detection model must be retrained from scratch whenever a new defect class emerges, which incurs significant time and computational cost. Incremental defect detection (IDD) enables models to continuously learn new defect classes without complete retraining, and has attracted increasing attention in industrial quality inspection [6].

Knowledge distillation is an effective strategy to achieve incremental defect detection [7,8]. During training, a pre-trained teacher model is utilized, and the outputs of its classification head, regression head, and feature layers serve as soft labels to guide the student model to align with the outputs of the teacher model. This enables the student model to learn new defect classes while retaining discriminative ability for old classes, thereby mitigating catastrophic forgetting. Xiong et al. [9] developed an adaptive dual-teacher incremental defect detection method, which incorporates both old-class and new-class teacher models. By utilizing a decoupled feature distillation mechanism, their method avoids inappropriate feature transfer and dynamically balances knowledge transfer between old and new defect classes, thus preserving the performance of old classes while improving the learning ability of new classes. Shen et al. [10] introduced class-similarity distillation and global-similarity distillation. The former adaptively assigns distillation weights based on the similarity between the prototypes of old and new classes, facilitating knowledge transfer across related categories. The latter maximizes mutual information between features of old and new classes to further strengthen cross-class feature correlation. This is further combined with an RPN-based sample assignment strategy to improve the stability and accuracy of incremental detection.

However, incremental defect detection methods based on knowledge distillation still encounter several challenges. First, global distillation tends to distribute gradient updates uniformly across regions, leading to insufficient distillation supervision on high-value regions, while the discriminative knowledge may be diluted by irrelevant information and noise during transfer. The ineffective alignment of low-value regions results in wasted gradient resources and hinders the student model’s ability to learn new classes. Secondly, industrial defect targets often possess ambiguous boundaries, and traditional regression distillation lacks effective mechanisms for filtering high-quality localization anchors, leading to low localization precision. Finally, defects exhibit significant variations in morphology and scale, resulting in different learning difficulties across categories. Existing models struggle to adaptively allocate learning resources, often overemphasizing easy-to-learn targets while underemphasizing hard-to-learn ones, which results in poor overall performance.

To address these issues, we propose an incremental defect detection model, SD-IDD, including high-confidence classification distillation, dual-stage cascaded regression distillation, and Intersection over Union (IoU)-driven difficulty-aware feature distillation. These selective distillation strategies enable dynamic selection and preferential resource allocation during the knowledge transfer process, ensuring that critical regions receive stronger distillation constraints while minimizing ineffective interference from low-value regions. Consequently, the model balances recognition capability between old and new classes and improves overall performance without relying on old class samples.

The main contributions of this research are summarized as follows:

(1) A high-confidence classification distillation strategy is developed, which distills only high-confidence anchors in the classification head. This strategy preserves semantic knowledge (e.g., texture priors) of similarity between old and new classes from the teacher model and effectively mitigates catastrophic forgetting.
(2) A dual-stage cascaded regression distillation strategy is proposed, which obtains a high-quality anchor set through geometric prior coarse filtering and statistical fine filtering. Integrated with IoU-weighted KL divergence distillation, this strategy substantially enhances the regression accuracy of the student model, forming a positive “filter-distill-accuracy enhancement” loop.
(3) An IoU-driven difficulty-aware feature distillation strategy is presented, which quantifies the regression difficulty of defect targets based on IoU and adaptively allocates distillation resources, prioritizing high-difficulty and critical feature regions. This strategy preserves the discriminative ability of old classes while enhancing the learning efficiency of new classes, thereby maintaining balanced recognition performance across classes.

## 2. Related Work

### 2.1. Defect Detection

Owing to the excellent feature self-learning capability, convolutional neural networks (CNNs) have become mainstream methods for industrial surface defect detection [11]. Object detection methods can be classified into single-stage and two-stage detectors. Single-stage detectors (e.g., the YOLO series [12,13,14,15], SSD [16], RetinaNet [17], and GFLv1 [18]) perform object classification and localization directly on the feature maps, resulting in faster detection speed. In contrast, two-stage detectors (e.g., R-CNN [19], Fast R-CNN [20], and Faster R-CNN [21]) first generate candidate regions likely to contain objects and then perform classification and localization, achieving higher detection accuracy. Li et al. [22] proposed a steel surface defect detection method based on YOLOX that integrates CSPCrossLayer, Shuffle Attention, and PSblock modules to enhance feature extraction and multi-scale fusion, achieving 77% mAP_50 on the NEU-DET dataset. Lu et al. [23] presented the WSS-YOLO method for steel surface defect detection, which integrates a WIoU-based dynamic non-monotonic focusing mechanism, C2f-DSC module, and GSConv with a VOVGSCSP structure into YOLOv8, achieving 82.3% mAP_50 on the NEU-DET dataset. Li et al. [24] developed a lightweight PCB defect detection method called HSD-YOLO, which adopts HGNetv2 as the backbone network and incorporates GSConv and DyHead structures. Compared with YOLOv8, HSD-YOLO improves mAP_50 by 0.6% on a public PCB defect dataset while substantially reducing the number of parameters. Deng et al. [25] introduced the EHIR method, formulating image registration in PCB defect detection as an edge-point-based energy optimization problem. By integrating the EET and EEDD modules, it achieves contour-consistency alignment and differential saliency localization, thereby maintaining high accuracy and strong robustness even under defect feature interference. Li et al. [26] designed a PCB defect detection network called DesNet, based on deformable convolutions. By deepening the network structure and enhancing feature extraction for high-level semantic representation, DesNet achieved a 3% improvement in mAP_50 over RetinaNet on the PCB defect detection dataset while meeting real-time detection requirements.

### 2.2. Incremental Object Detection

Incremental learning aims to enable models to continuously obtain new knowledge while retaining previously learned knowledge, without the necessity of retraining the entire model [27]. Within the incremental learning paradigm, class-incremental learning focuses exclusively on learning to classify both newly introduced and previously learned classes [28], whereas incremental object detection further extends the scope of the task by simultaneously performing object localization in addition to classification [29]. Early research mainly focused on class-incremental learning [30,31,32]. Nonetheless, recent findings indicate that merely classifying new and old classes is insufficient for practical applications, as accurate localization of both new and old objects is also required. As a result, incremental object detection has emerged as a research hotspot [33].

Incremental object detection methods based on knowledge distillation adopt a teacher–student framework during the training of new classes, where the knowledge of the old model is transferred into the new model in the form of soft labels or feature alignment, thereby mitigating catastrophic forgetting [34]. Shmelkov et al. [35] presented a dual distillation mechanism for classification and regression in Fast R-CNN, which preserves the output distribution of the teacher model on new class data, thereby enabling incremental detection without old class samples. Hao et al. [36] developed the CIFRCN method, which incorporates RPN domain expansion, multi-layer distillation in the FRCN branch, and a nearest-prototype classifier in Faster R-CNN, thereby achieving end-to-end incremental detection using only new class data. Can et al. [37] presented the Faster ILOD framework, which introduces multi-network adaptive distillation in Faster R-CNN, jointly preserving knowledge from feature layers, RPN outputs, and RCN outputs. Chen et al. [38] incorporated multi-level feature distillation and a reweighting strategy in the FPN framework to better preserve old class knowledge across multi-scale features. Liu et al. [39] proposed an incremental detection method in Transformer-based architectures, which combines detector knowledge distillation with experience replay to maintain knowledge retention. Feng et al. [40] proposed an incremental detection method named Elastic Response Distillation, which sets thresholds based on the statistical characteristics of responses in the classification head and regression head to filter high-quality positive sample response anchors, thus realizing the accurate transfer of key knowledge of old classes.

Although existing methods have achieved some success in mitigating catastrophic forgetting, incremental object detection continues to encounter several challenges. First, most current distillation strategies rely on global alignment and fail to differentiate between high-value and low-value regions, resulting in discriminative knowledge being weakened by noise interference during transfer. To address this issue, we propose a high-confidence classification distillation strategy, which effectively retains critical discriminative knowledge and reduces noise interference by distilling only high-confidence anchors. Secondly, regression distillation often struggles to effectively filter high-quality anchors, which significantly hampers the improvement of localization accuracy in the presence of fuzzy defect boundaries. In response, this study designs a dual-stage cascaded regression distillation strategy, utilizing geometric prior coarse filtering and statistical fine filtering mechanisms to ensure the precise transfer of localization knowledge. Although Feng et al. [40] perform selective distillation based on responses, it adopts a unified selection strategy for both the classification head and regression head. In contrast, we implement targeted strategies for the classification head and regression head, respectively, leading to more precise and efficient knowledge transfer. Finally, defect targets display complex morphologies and significant scale variations, with learning difficulty differing across categories; the lack of targeted resource allocation mechanisms leads models to overlook hard-to-learn targets. Consequently, we introduce an IoU-driven difficulty-aware feature distillation strategy, which achieves adaptive learning and prioritizes resources for high-difficulty targets, thereby balancing the feature learning capabilities of old and new classes.

## 3. Methods

### 3.1. Overall Architecture

**Figure 1:**
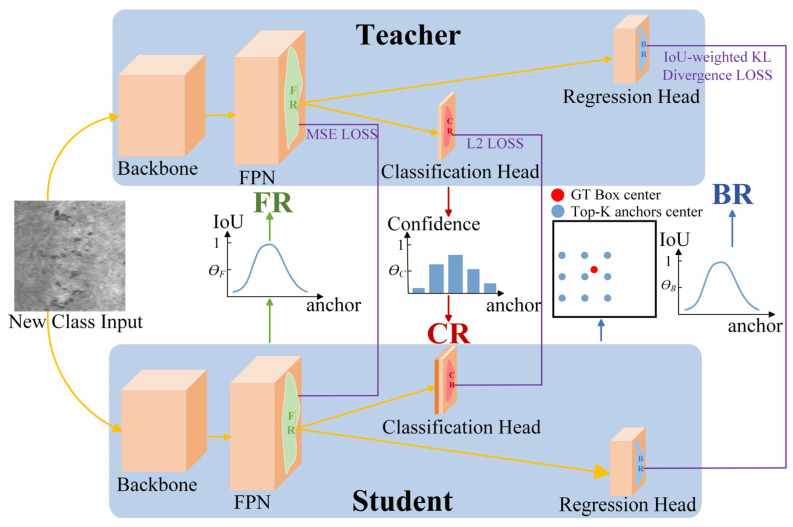
SD-IDD network architecture.

We propose an incremental defect detection model called SD-IDD (Selective Distillation for Incremental Defect Detection), whose network architecture is illustrated in Figure 1. The training process of SD-IDD adheres to the classical incremental learning paradigm. During the incremental training phase, SD-IDD requires only the model weights obtained from the previous training phase (i.e., the teacher model) and the images and annotations of the new classes to be learned in the current phase. Then the student model can learn both the old classes already mastered by the teacher model and the new classes in the current phase. Both the teacher model and the student model of SD-IDD adopt the GFLv1 structure, which comprises a backbone network, FPN, a classification head, and a regression head. The sole distinction between the teacher model and the student model lies in the number of class channels in the classification head. The classification head of the teacher model has a number of class channels equal to the number of old classes, whereas the classification head of the student model has a number of class channels equal to the sum of the old and new classes.

We propose three selective distillation strategies at different components of SD-IDD. These strategies aim to filter only critical knowledge for distillation, thereby reducing noise interference and effectively mitigating catastrophic forgetting. Specifically, a high-confidence classification distillation strategy is proposed for the classification head of SD-IDD. This strategy obtains the confidence distribution of new-class samples over the old-class categories from the teacher model’s classification head outputs. Then, a threshold is set to retain only the high-confidence anchors as the classification head distillation region (CR). Furthermore, a dual-stage cascaded regression distillation strategy is proposed for the regression head of SD-IDD. First, the Top-K anchor boxes closest to the center point of the Ground Truth Boxes (GT Boxes) are selected as the candidate set. Subsequently, a threshold is set on the IoU distribution of the candidate set, and anchors with IoU values above the threshold are designated as the regression head distillation region (BR). In addition, an IoU-driven difficulty-aware feature distillation strategy is proposed for the FPN of SD-IDD. The regression difficulty of each target is quantified based on the IoU value, and a threshold is applied to select anchors with higher regression difficulty as the FPN feature distillation region (FR).

Therefore, the total loss of the SD-IDD student model is as follows:

(1) $$L_{all}=\lambda_{1}L_{GFL}+\lambda_{2}L_{cls}\left(C_{T},C_{SO}\right)+\lambda_{3}L_{bbox}\left(B_{T},B_{S}\right)+\lambda_{4}L_{FPN}\left(F_{T},F_{S}\right)$$

where $\lambda_{i}$ denotes the weight coefficient for each loss term, $\lambda_{1}=2$, and $\lambda_{2}=\lambda_{3}=\lambda_{4}=1$. C, B, and F denote the outputs of the classification head, regression head, and FPN feature layers, respectively. T and S denote the teacher model and the student model, respectively. The definitions of all symbols in Equation (1) are provided in Table 1. The first loss term $L_{GFL}$ corresponds to the original training loss of the student model. The remaining three terms correspond to the distillation losses applied to the student model, where $L_{cls}$ is the L2 distillation loss on the classification head distillation region (CR), $L_{bbox}$ is the IoU-weighted KL divergence distillation loss on the regression head distillation region (BR), and $L_{FPN}$ is the MSE distillation loss on the FPN feature distillation region (FR). Detailed descriptions of these three distillation losses are provided in the following subsections.

### 3.2. High-Confidence Classification Distillation Strategy

Global classification distillation suffers from severe noise interference and knowledge dilution. Therefore, we propose a high-confidence classification distillation strategy, which filters high-confidence anchors and performs classification distillation exclusively in semantic confusion regions, thereby preserving the discriminative knowledge of old classes while minimizing ineffective alignment in low-value regions.

During the training process on old classes, the teacher model has already learned discriminative features, but this knowledge is not uniformly distributed. Numerous background region anchors yield low-confidence predictions when the student model is trained on new class samples, as both the old class probabilities and the IoU values are close to 0. If distillation is conducted in such regions, it will introduce ambiguous semantic signals, hindering the student model’s learning of new classes. Conversely, within the foreground regions of new class samples, the student model tends to misclassify a new class as a similar old class. In this case, the predicted probability of the old class is relatively high, and the IoU with the GT Box is close to 1, resulting in high-confidence predictions. These high-confidence predictions of old classes encode the feature similarity and texture priors between old and new classes. By enforcing alignment on such responses, the student model can maintain consistent class boundaries with the teacher model in semantic confusion regions, thereby preserving the learned feature patterns and preventing gradients from newly learned classes from disrupting the existing feature space.

Based on the above idea, we design the high-confidence classification distillation strategy illustrated in Algorithm 1. Let *oc* denote the number of old classes, *nc* denote the number of new classes, *N* denote the number of anchors, and *t* denote the temperature factor for softening. Then, the output of the teacher model’s classification head is $C_{T}\in R^{N\times oc}$, and the output of the student model’s classification head is $C_{S}=C_{SO}+C_{SN}\in R^{N\times \left(oc+nc\right)}$. First, softmax normalization is applied to the output of the teacher model’s classification head along the class channel dimension. The maximum value along the class channel dimension is then taken as the confidence score of each anchor:

(2) $$C=\max\left(\mathrm{softmax}\left(\frac{C_{T}}{t}\right)\right)$$

where t=2.

Next, the mean $\mu_{C}$ and standard deviation $\sigma_{C}$ of the confidence score distribution C are computed, and an adaptive threshold that dynamically adjusts according to the sample characteristics is formulated as:

(3) $$\theta_{C}=\mu_{C}+\alpha \sigma_{C}$$

where α is a scaling factor and is set to 2.

The set of anchors whose confidence scores exceed the threshold $\theta_{C}$ is denoted as CR. On CR, the L2 loss between the output of the teacher model’s classification head and the outputs corresponding to the old-class channels in the student model’s classification head is calculated. Therefore, the distillation loss function for the classification head is as follows:

(4) $$L_{cls}\left(C_{T},C_{SO}\right)={\sum_{i\in CR}\left(C_{T}^{i}-C_{So}^{i}\right)}^{2}$$

By aligning the classification predictions of the teacher and student models on high-confidence anchors, this loss function preserves the student model’s ability to recognize old classes and distinguish between old and new classes, effectively mitigating catastrophic forgetting.
**Algorithm 1:** High-confidence classification distillation strategy**Input:** The prediction results of new classes samples on the teacher model’s classification head $C_{T}$ **Output:** Classification head distillation region CR **Variable:** The number of anchors N, the i-th anchor $A_{i}$, scaling factor α 1: Compute $C=max\left(softmax\left(C_{T}/t\right)\right)$ 2: Compute $\mu_{C}=mean\left(C\right)$ 3: Compute $\sigma_{C}=std\left(C\right)$ 4: Set threshold $\theta_{C}=\mu_{C}+\alpha \sigma_{C}$ 5: **for** i=1 **to** N **do** 6:       **if** $C_{i}>\theta_{C}\;$**then** 7:               $A_{i}\in CR$ 8: **return** CR

### 3.3. Dual-Stage Cascaded Regression Distillation Strategy

To effectively filter out noisy anchors and enhance localization accuracy under fuzzy defect boundaries, we propose a dual-stage cascaded regression distillation strategy. This strategy adopts a two-level mechanism of “geometric prior coarse filtering + statistical fine filtering”, combined with IoU-weighted KL divergence distillation, to achieve precise transfer of localization knowledge from high-quality anchors. This strategy reduces the interference caused by ineffective alignment while improving the localization accuracy of the student model.

Traditional regression distillation methods directly align all anchors, yet a single image often generates tens of thousands of anchors, among which only a very small fraction actually contain the target. Such indiscriminate distillation introduces a large number of low-IoU noisy predictions, which hampers the student model’s learning of critical localization knowledge. Meanwhile, defect boundaries in industrial scenarios tend to be fuzzy. If L2 loss is applied to distill defect coordinates, slight discrepancies between the teacher and student models at fuzzy boundaries will be magnified by the squared error, resulting in gradient instability. To address these issues, we combine GFLv1’s probabilistic distribution regression mechanism with IoU-weighted KL divergence distillation, thereby fully transferring the fuzzy boundary information encoded in the teacher model’s predictions. Moreover, through dual-stage filtering, the strategy ensures that only high-value regression knowledge from selected anchors is transferred, thereby enhancing the regression accuracy of the student model.

The dual-stage cascaded regression distillation strategy proposed in this section is detailed in Algorithm 2. In the first stage of geometric prior coarse filtering, based on the spatial clustering characteristics of defect targets, for each GT Box, the Top-K anchors closest to its center are selected to form a candidate set covering the core region of the defect target. In the second stage of statistical fine filtering, the mean $\mu_{B}$ and standard deviation $\sigma_{B}$ of the IoU distribution in the candidate set are computed, and an adaptive threshold is constructed:

(5) $$\theta_{B}=\mu_{B}+\beta \sigma_{B}$$

where β is a scaling factor set to 2.

Anchors with IoU values exceeding the threshold $\theta_{B}$ constitute the regression head distillation region BR. On BR, IoU-weighted KL divergence distillation is applied to enable precise transfer of regression knowledge from high-quality anchors. Specifically, softmax is performed on the teacher model’s regression head output $B_{T}$, and log-softmax is conducted on the student model’s regression head output $B_{S}$, followed by IoU-weighted KL divergence distillation. Therefore, the distillation loss function for the regression head is as follows:

(6) $$L_{bbox}\left(B_{T},B_{S}\right)=\frac{1}{\left|BR\right|}\sum_{j\in BR}IoU\left(j\right)\times D_{KL}\left(\mathrm{softmax}\left(\frac{B_{T}}{t}\right)∥\log\_\mathrm{softmax}\left(\frac{B_{S}}{t}\right)\right)$$

where |BR| denotes the number of anchors in BR, and $IoU\left(j\right)$ represents the IoU value between the anchor *j* and its matched GT Box.
**Algorithm 2:** Dual-stage cascaded regression distillation strategy**Input:** The bounding box coordinates of all anchors predicted by the student model, the bounding box coordinates of all GT Boxes **Output:** Regression head distillation region BR **Variable:** The number of anchors N, the i-th anchor $A_{i}$, scaling factor β **Stage 1:** 1: Compute the IoU matrix between all anchors and all GT Boxes 2: Compute the IoU vector between every anchor and the GT Box matching the anchor I 3: Compute the distances between the anchors’ center point and the GT Boxes’ center point 4: Take the Top-K anchors closest to every GT Box’s center point m **Stage 2:** 5: Compute $\mu_{B}=mean\left(I_{m}\right)$ 6: Compute $\sigma_{B}=std\left(I_{m}\right)$ 7: Set threshold $\theta_{B}=\mu_{B}+\beta \sigma_{B}$ 8: **for** i=1 **to** N **do** 9:       **if** $I_{i}>\theta_{B}\;$**then** 10:              $A_{i}\in BR$ 11: **return** BR

The dual-stage cascaded regression distillation strategy, combined with IoU-weighted KL divergence distillation, establishes a self-enhancing knowledge transfer loop. First, the dual-stage filtering ensures that only high-IoU anchors containing valid information are distilled, thereby avoiding the interference of large amounts of noise and useless knowledge. Subsequently, the KL divergence transfers precise probability distributions, enabling the student model to inherit the distributional characteristics of the teacher model. The IoU weighting further ensures that high-IoU anchors dominate gradient updates. The improvement in the student model’s localization ability in turn enhances the quality of filtering in subsequent iterations, thus forming a positive feedback loop of “filter–distill–accuracy enhancement”.

### 3.4. IoU-Driven Difficulty-Aware Feature Distillation Strategy

To tackle the dilemma in traditional feature distillation between “over-distillation suppressing new class learning” and “under-distillation causing catastrophic forgetting” in incremental defect detection, we propose an IoU-driven difficulty-aware feature distillation strategy. This strategy quantifies the regression difficulty of defect targets using IoU and employs a dynamic threshold mechanism to adaptively allocate distillation resources, thereby focusing more attention on difficult targets. In this approach, it preserves old class features while leaving room for learning new class features, thereby avoiding rigidity in the feature space.

In contrast to methods that rely solely on fixed distillation intensity, this strategy dynamically adjusts resource allocation based on the learning difficulty of each target. When the maximum matched IoU of a target is high, it suggests that the target is easy to regress. In such cases, the threshold should be raised, preserving only a limited number of high-quality anchors for distillation. When the maximum matched IoU is low, it indicates that the target is difficult to regress. In this situation, the threshold should be lowered to increase the number of distillation anchors and guide the student model to focus more on the features of difficult samples. In this way, a biased allocation of distillation resources is achieved.

The IoU-driven difficulty-aware feature distillation strategy proposed in this section is illustrated in Algorithm 3. First, the IoU matrix between all anchors and all GT Boxes is computed. For each anchor box, the maximum IoU value with its matched GT Box is taken to form a vector *I*. For each GT Box, the maximum IoU value among all anchors matched to it is taken to form a vector *G*. Then, an adaptive quality filtering threshold vector is constructed as follows:

(7) $$\theta_{F}=\gamma ⊗G$$

where γ is a learnable adaptive scaling factor constrained to the range (0, 1). ⊗ denotes element-wise multPythoniplication.

The set of distillation anchors obtained through threshold $\theta_{F}$ is denoted as FR. On FR, MSE loss distillation is performed on the output $F_{T}$ of the teacher model’s FPN feature layer and the output $F_{S}$ of the student model’s FPN feature layer. Therefore, the distillation loss function for the FPN feature layer is as follows:

(8) $$L_{FPN}\left(F_{T},F_{S}\right)=\frac{1}{\left|FR\right|}{\sum_{k\in FR}\left(F_{T}^{k}-F_{S}^{k}\right)}^{2}$$

where |FR| represents the number of anchor boxes in FR. This strategy can dynamically select the most discriminative feature regions during the distillation process, ensuring that limited resources are prioritized for difficult targets. Consequently, it achieves a balance between retaining old knowledge and learning new knowledge, significantly enhancing the overall performance of the model in incremental detection tasks.
**Algorithm 3**: IoU-driven difficulty-aware feature distillation strategy**Input:** The bounding box coordinates of all anchors predicted by the student model, the bounding box coordinates of all GT Boxes **Output:** FPN feature distillation region FR **Variable:** The number of GT Boxes M, the number of anchors N, the i-th anchor $A_{i}$, GT Box index j, adaptive scaling factor γ **Operator:** Element-wise multiplication ⊗ 1: Compute the IoU matrix between all anchors and all GT Boxes $D\in R^{N\times M}$ 2: Compute the IoU vector between every anchor and the GT Box matching the anchor $I\in R^{N}$ 3: Compute every GT Box’s IoU max vector $G\in R^{M}$ 4: Set threshold vector $\theta_{F}=\gamma ⊗G\in R^{M}$ 5: **for** j=1 **to** M **do** 6:        **for** i=1 **to** N **do** 7:             compute index j by $I_{i}==D_{\left(i,j\right)}$ 8:             **if** $I_{i}>\;\theta_{Fj}\;$**then** 9:                   $A_{i}\in FR$ 10: **return** FR

## 4. Experiments

We carried out experiments on the public defect datasets NEU-DET [41] and DeepPCB [42] to assess the effectiveness of the SD-IDD model.

### 4.1. Experimental Settings

Both the teacher model and the student model of SD-IDD utilized the GFLv1 architecture, with ResNet50 as the backbone network and FPN as the neck. All experiments were executed on an RTX 4090D GPU with 24GB memory, running on Ubuntu version 20.04, Python version 3.8.10, PyTorch version 1.11.0, and CUDA version 11.3. The experiments were conducted on the mmdetection 3.0.0 platform. In the training process, the batch size was set to 2, the number of epochs was 24, and SGD was employed as the optimizer, with an initial learning rate of 0.001, a momentum of 0.9, and a weight decay of 0.0001. For the learning rate schedule, two strategies were employed: Linear Learning Rate Decay (LinearLR), where the learning rate starts at 0.001 and decays linearly, reaching 0.001 times the initial value after 500 iterations, and Multi-Step Learning Rate Scheduling (MultiStepLR), where the learning rate is multiplied by 0.1 at the 16th and 22nd epochs, gradually reducing the learning rate during the later stages of training to help the model converge better. Other experimental settings followed the default configurations of the MMDetection 3.0.0 platform.

### 4.2. Datasets and Evaluation Metrics

The NEU-DET dataset is a defect dataset on hot-rolled steel strips, containing six defect types: crazing, inclusion, patches, pitted_surface, rolled-in_scale, and scratches. The DeepPCB dataset is a defect dataset of PCB boards, with six defect types: open, short, mousebite, spur, copper, and pin-hole. In this section, the first three classes were learned in the initial phase, and the last three classes were learned in the incremental phase. The detailed class division of the NEU-DET and DeepPCB datasets during the initial and incremental phases is detailed in Table 2.

To evaluate the performance of an incremental object detection model, it is essential to assess its ability to learn new knowledge and its ability to avoid catastrophic forgetting of old knowledge. Accordingly, this section evaluated the SD-IDD model along three dimensions: mAP_50 for old classes, new classes, and all classes, to measure the model’s stability, plasticity, and overall performance, which are denoted as mAP_old, mAP_new, and mAP_all, respectively.

### 4.3. Ablation Experiments

To verify the effectiveness and synergy of the three distillation strategies proposed in this research, ablation experiments were carried out on NEU-DET and DeepPCB. The results are presented in Table 3 and Table 4. In the tables, “None” indicates that no distillation loss is applied to the classification head, regression head, or FPN. “All” signifies that global classification distillation loss, global regression distillation loss, and global feature distillation loss are applied to the classification head, regression head, and FPN, respectively. “CR” denotes the implementation of the high-confidence classification distillation strategy on the classification head. “BR” indicates the use of the dual-stage cascaded regression distillation strategy on the regression head. “FR” represents the use of the IoU-driven difficulty-aware feature distillation strategy on the FPN.

(1) Experiments on the NEU-DET Dataset

When no distillation loss was applied to the classification head, regression head, or FPN, mAP_old was 17.3%, mAP_new was 68.1%, and mAP_all was 42.7%. This suggests that without distillation constraints, the detection performance for new classes was relatively good, but the performance for old classes had severely degraded, showing clear signs of catastrophic forgetting.

As shown in Rows 2, 3, and 8 of Table 3, when no classification distillation was applied to the classification head, yet the SD-IDD distillation strategies were applied to the regression head and FPN, mAP_all was 56.9%. When global classification distillation was performed on the classification head, mAP_all increased to 61.2%. When the high-confidence classification distillation strategy was applied to the classification head, mAP_all further rose to 63.6%. The high-confidence classification distillation strategy surpassed global classification distillation by 2.4%, demonstrating that distillation on a large number of background anchors and low-confidence anchors in the classification head hampers the student model’s learning. In contrast, the high-confidence strategy compels the student model to retain old class responses on high-confidence anchors, effectively filtering semantic noise from low-confidence anchors.

As illustrated in Rows 4, 5, and 8 of Table 3, when the SD-IDD distillation strategies were applied to the classification head and FPN, using global regression distillation loss on the regression head improved mAP_all by 3.6% compared to using no regression distillation, whereas applying the dual-stage cascaded regression distillation strategy on the regression head further enhanced mAP_all by an additional 2.3% compared to the global regression distillation loss. This suggests that global regression distillation loss also introduces noise, whereas the dual-stage cascaded regression distillation strategy reduces regression noise from low-IoU anchors by focusing on IoU-weighted KL divergence distillation over high-IoU anchors, thereby improving the student model’s regression accuracy.

As depicted in Rows 6, 7, and 8 of Table 3, when SD-IDD distillation strategies were applied to the classification and regression heads, using global feature distillation loss on the FPN increased mAP_all by 3.2% compared to not using feature distillation loss, and using the IoU-driven difficulty-aware feature distillation further improved mAP_all by 1.7% over the global feature distillation loss. This indicates that the IoU-driven difficulty-aware feature distillation effectively reduces interference from low-value regions, concentrating the distillation on salient features. When all three SD-IDD distillation strategies were applied concurrently, mAP_old, mAP_new, and mAP_all reached their maximum values of 58.2%, 69%, and 63.6%, respectively, representing improvements of 40.9%, 0.9%, and 20.9% over the catastrophic forgetting. This not only substantiates the effectiveness of each distillation strategy individually but also illustrates their strong synergy in collectively enhancing performance, thereby preventing catastrophic forgetting of old classes while bolstering the learning capability for new classes.

(2) Experiments on the DeepPCB Dataset

When no distillation loss was applied to the classification head, regression head, or FPN, mAP_old was 30.5%, mAP_new was 94.9%, and mAP_all was 62.7%. This further indicates that in the absence of distillation constraints, the learning of new classes severely overwrites the knowledge of old classes, leading to significant catastrophic forgetting. Adding global distillation loss to the classification head, regression head, and FPN improves mAP_all by 11%, 9.1%, and 9.3%, respectively, compared to the corresponding parts with no distillation loss. Meanwhile, the three distillation strategies of SD-IDD improve mAP_all by 2.6%, 2.3%, and 1.9%, respectively, compared to the global distillation loss applied to the corresponding parts. This further confirms that the large number of background and low-quality anchors in new class samples constitutes distillation noise. Performing indiscriminate distillation across all anchors introduces a significant amount of noise into the student model’s learning process, which in turn harms performance. When all three SD-IDD distillation strategies were applied simultaneously on the DeepPCB dataset, mAP_old, mAP_new, and mAP_all reached their maximum values of 99.3%, 97.3%, and 98.3%, respectively, reflecting improvements of 68.8%, 2.4%, and 35.6% over catastrophic forgetting. Through its three distillation strategies, SD-IDD enables the student model to focus on learning core knowledge while suppressing noise, achieving more accurate knowledge transfer and significant performance gains.

(3) Hyper-parameter Experiments on the NEU-DET Dataset

To further investigate the robustness of SD-IDD, a series of hyperparameter experiments was conducted on the NEU-DET dataset under the same incremental setting as described in Table 2. All experiments strictly maintained identical backbone architecture, training schedule, and data split to ensure fairness. The evaluation metric remained mAP_old, mAP_new, and mAP_all.

To evaluate the influence of the scaling factors α and β on the performance of the SD-IDD model, ablation experiments were conducted on the NEU-DET dataset, and the results are shown in Table 5. The scaling factor α controls the strictness of the confidence threshold in the high-confidence classification distillation strategy, while β regulates the filtering strength of the IoU threshold in the dual-stage cascaded regression distillation strategy. Larger values of α or β result in stricter anchor selection, thereby reducing the participation of low-quality anchors in the distillation process. As shown in Table 5, under different combinations of α and β, the overall performance of the SD-IDD model remains stable, with mAP_all fluctuating only within a small range (63.2–63.6%). The best overall performance is achieved when both α and β are set to 2, indicating that moderately stricter filtering is beneficial for effective knowledge transfer in both classification and regression. The limited performance variation across different settings demonstrates that the proposed high-confidence classification distillation strategy and dual-stage cascaded regression distillation strategy exhibit good stability.

To evaluate the effectiveness of the learnable adaptive scaling factor γ in the IoU-driven difficulty-aware feature distillation strategy, experiments were conducted on the NEU-DET dataset, as shown in Table 6. Specifically, we compared fixed γ values (0.5 and 0.8) with a learnable adaptive γ constrained within the range of 0 to 1. The experimental results indicate that, compared with the adaptive setting, fixing γ leads to a slight decline in overall performance. When γ=0.5 and γ=0.8, the mAP_all values are 63.1% and 62.9%, respectively, whereas the adaptive γ achieves the best overall performance of 63.6%. This suggests that manually setting γ may limit the flexibility of the threshold across different samples. In contrast, a learnable γ can dynamically adapt to the IoU distribution during training and allocate knowledge transfer resources more reasonably, thereby improving the overall detection performance.

To analyze the effect of the Top-K in the dual-stage cascaded regression distillation strategy, experiments were conducted on the NEU-DET dataset, and the results are shown in Table 7. Top-K controls the number of candidate anchors selected for each GT Box during the first-stage geometric prior coarse filtering, thereby determining the sample size used for the subsequent statistical fine filtering. The experimental results show that when Top-K increases from 80 to 100, mAP_all improves from 63.0% to 63.6%, indicating an overall upward trend in performance. However, when Top-K is further increased to 120, mAP_all decreases to 63.2%. This suggests that a small Top-K limits the number of candidate anchors, causing some potentially high-quality samples to be prematurely discarded before statistical filtering. In contrast, an excessively large Top-K introduces more low-quality anchors, weakening the effectiveness of the second-stage statistical filtering and increasing noise interference. The best performance is achieved when Top-K is set to 100, demonstrating that a moderate candidate scale can achieve a better balance between covering the core region of targets and suppressing noise.

### 4.4. Comparative Experiments

**Figure 2:**
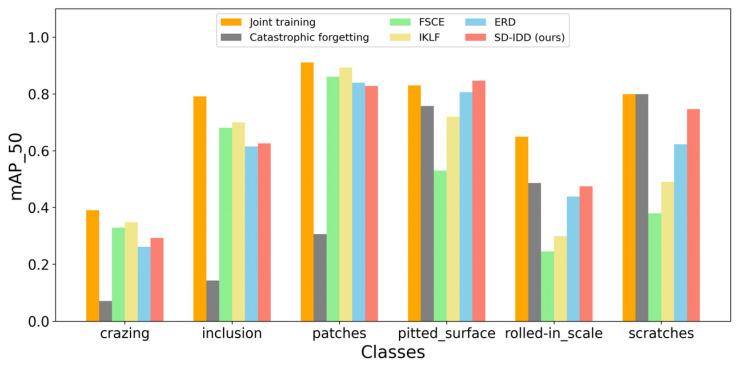
The mAP_50 of each class on NEU-DET for different methods.

**Figure 3:**
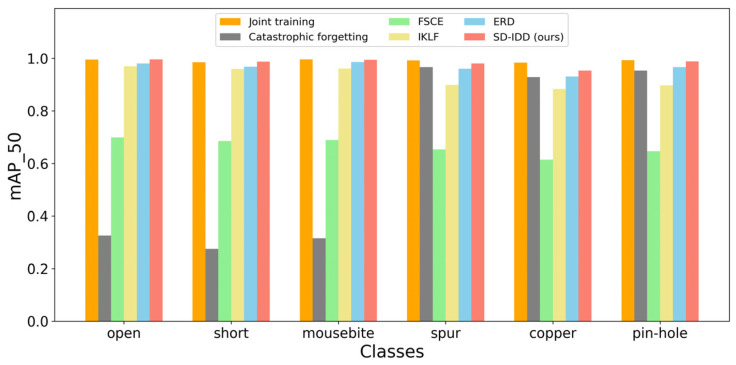
The mAP_50 of each class on DeepPCB for different methods.

Table 8 and Table 9 present the comparative results of the SD-IDD model on NEU-DET and DeepPCB, respectively, against the joint training method, fine-tuning method, and several state-of-the-art incremental object detection methods, including FSCE [43], IKLF [44], and ERD [40]. Specifically, the experimental data for FSCE and IKLF were sourced from reference [44], and the results for ERD were reproduced by this research on NEU-DET and DeepPCB. Figure 2 and Figure 3 illustrate the mAP_50 for each class on NEU-DET and DeepPCB, respectively, for all compared methods.

To ensure a fair comparison, all compared methods were implemented under the same experimental settings as SD-IDD, including the same backbone network ResNet50, training epochs, batch size, optimizer configuration, learning rate schedule, evaluation metrics, and hardware environment. All methods adhered strictly to the incremental class division setup outlined in Table 2.

Joint training refers to training all classes at once on GFLv1 and is considered the upper bound of incremental object detection. In contrast, fine-tuning refers to training directly on the new class samples using the model weights of old classes, which is susceptible to catastrophic forgetting and is regarded as the lower bound of incremental object detection. On NEU-DET, mAP_old decreased sharply from 69.8% (joint training) to 17.3% (fine-tuning), showing a decline of 52.5%. On DeepPCB, mAP_old plummeted from 99.3% to 30.5%, showing a decrease of 68.8%. Although the training data consisted of new class samples, the mAP_new value was also lower than that of joint training due to the impact of old class model weights. Compared to the fine-tuning method, SD-IDD achieved improvements of 40.9%, 0.9%, and 20.9% in mAP_old, mAP_new, and mAP_all on NEU-DET, respectively, and improvements of 68.8%, 2.4%, and 35.6% on DeepPCB. These results indicate that SD-IDD effectively alleviates the issue of catastrophic forgetting.

On NEU-DET, IKLF exhibited the best performance on old classes, followed by FSCE, achieving mAP_old of 64.7% and 62.3%, respectively. However, both methods performed poorly on the new classes, with mAP_new of only 50.3% and 38.5%. In contrast, SD-IDD achieved the highest performance on the new classes, followed by ERD, attaining mAP_new of 69.0% and 62.2%, respectively. However, both SD-IDD and ERD performed worse than IKLF and FSCE in terms of old class performance, achieving mAP_old of only 58.2% and 57.2%. While SD-IDD and ERD are based on the single-stage object detector GFLv1, IKLF and FSCE are based on the two-stage object detector Faster R-CNN. Two-stage detectors generally exhibit slower detection speed but higher detection accuracy compared to single-stage detectors, which is particularly evident on complex and irregular defect datasets such as NEU-DET. Furthermore, the structural complexity of two-stage object detectors hinders gradient updates, making them more likely to maintain the status quo during incremental learning, and thus less effective in learning new classes. However, considering that industrial defect detection necessitates high real-time performance, SD-IDD leverages a lightweight single-stage architecture to achieve mAP_all of 63.6%, significantly surpassing that of ERD (59.7%), IKLF (57.5%), and FSCE (50.4%), thereby making it more suitable for industrial defect detection scenarios.

The superiority of SD-IDD was more outstanding on the DeepPCB dataset. It achieved mAP_all of 98.3%, which is only 0.8% lower than that of joint training (99.1%), and significantly surpassed that of ERD (96.5%), IKLF (92.8%), and FSCE (66.4%). Remarkably, SD-IDD achieved mAP_old of 99.3%, comparable to joint training, while also maintaining a high mAP_new of 97.3%. This suggests that, on a defect dataset with regular patterns like PCB, the distillation strategies of SD-IDD almost completely overcome catastrophic forgetting, perfectly balancing the ability to retain old class knowledge and learn new class knowledge.

**Figure 4:**
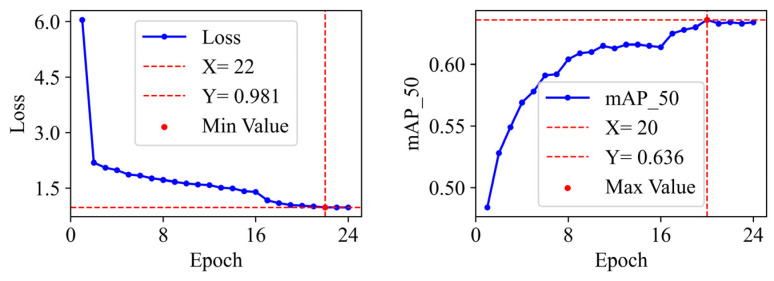
The training loss curve and mAP_50 curve of SD-IDD on the NEU-DET dataset.

**Figure 5:**
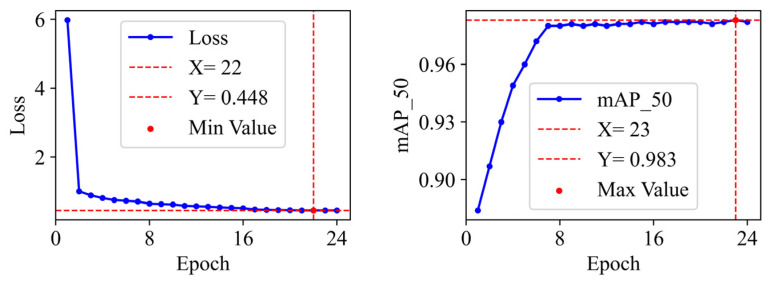
The training loss curve and mAP_50 curve of SD-IDD on the DeepPCB dataset.

Figure 4 and Figure 5 illustrate the curve of training total loss and mAP_50 with respect to the number of training epochs for SD-IDD on the NEU-DET and DeepPCB datasets, respectively. As depicted in Figure 4, the total loss of SD-IDD on NEU-DET exhibited a clear downward trend throughout the training progress, ultimately converging at its minimum loss value of 0.981 at epoch 22. Concurrently, the mAP_50 steadily increased, reaching its peak of 0.636 at epoch 20. In Figure 5, it can be observed that with further training, the total loss of SD-IDD on DeepPCB continuously decreased and eventually stabilized, converging at its lowest value of 0.448 at epoch 22. The mAP_50 continuously increased, attaining its highest value of 0.983 at epoch 23.

### 4.5. Visualization

**Figure 6:**
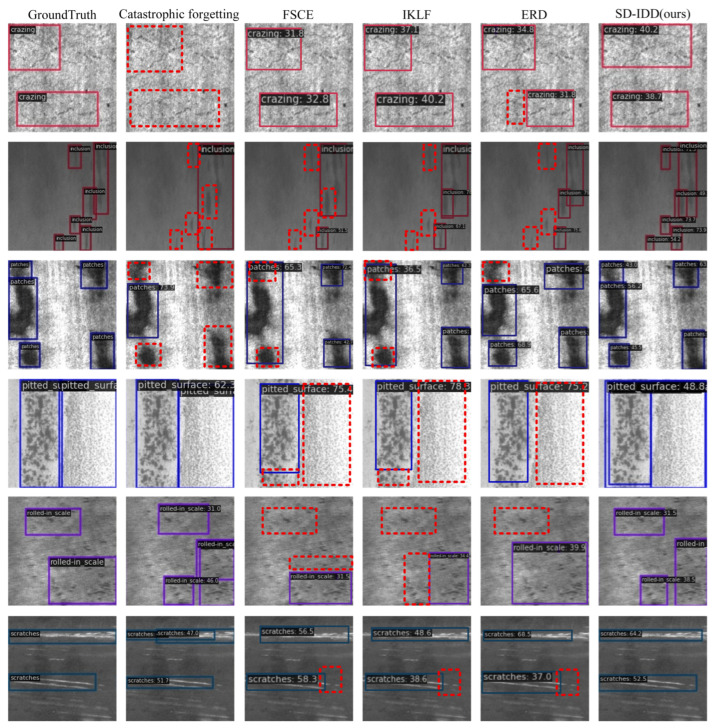
Partial detection results on the NEU-DET dataset.

Figure 6 illustrates the partial detection results of SD-IDD, Catastrophic forgetting, FSCE [43], IKLF [44], and ERD [40] on the NEU-DET dataset. Rows 1 to 6 correspond to different defect classes: crazing, inclusion, patches, pitted_surface, rolled-in_scale, and scratches, respectively. The first column presents the Ground Truth images. The second column shows the detection results of catastrophic forgetting caused by the fine-tuning method. The third, fourth, fifth, and sixth columns display the detection results of FSCE, IKLF, ERD, and the proposed SD-IDD method, respectively. The red dashed boxes highlight the missed detections and inaccurate localizations produced by the comparison methods.

As shown in the second column, the fine-tuning method indeed causes catastrophic forgetting: it performs extremely poorly on old classes while achieving relatively good performance on new classes. Specifically, the fine-tuning method fails to detect crazing defects, indicating a complete forgetfulness of the feature for this category. For inclusion defects, it only identifies the approximate regions but cannot accurately detect the number or precise locations of defects, resulting in very low regression accuracy. For patch defects, it detects only the most salient one and misses four defects. In contrast, the proposed SD-IDD method accurately detects the number and location of all old-class defects, demonstrating that it effectively alleviates catastrophic forgetting.

In Row 2, the inclusion defects are small, with fuzzy boundaries, dense distribution, and high adhesiveness. FSCE, IKLF, and ERD misidentify 3 defects in the upper-right area as 1 or 2 defects, and all methods failed to detect the two less noticeable defects in the lower-left region. Only SD-IDD perfectly detected all 6 inclusion defects, with predicted boxes highly overlapping with the GT Boxes. In Row 3, the patches on the left contain 3 defect targets. FSCE and IKLF recognized them as a single defect, whereas ERD detected 2 out of 3. Only SD-IDD successfully detected all 3 defects. In Row 4, pitted_surface defects are characterized by dense, uneven, spot-like distributions. The defect on the right is lightly colored and finely granulated, making it easily mistaken for the background. FSCE, IKLF, and ERD all failed to detect it, whereas only SD-IDD successfully identified it. In Row 5, FSCE, IKLF, and ERD overlooked the rolled-in_scale defect in the upper-left corner, whereas SD-IDD detected it. In Rows 1 and 6, all methods detected the crazing and scratches defects, but SD-IDD exhibited higher confidence levels.

Overall, FSCE, IKLF, and ERD experienced obvious missed detections on inclusion, patches, pitted_surface, and rolled-in_scale defects, and demonstrated lower confidence on crazing and scratches defects. In contrast, the proposed SD-IDD method perfectly detected all defect targets, with higher confidence and more accurate bounding box localization. Notably, for the challenging inclusion defects, the predicted boxes from SD-IDD showed considerable overlap with the GT Boxes, effectively reducing background noise interference and exhibiting superior regression accuracy.

**Figure 7:**
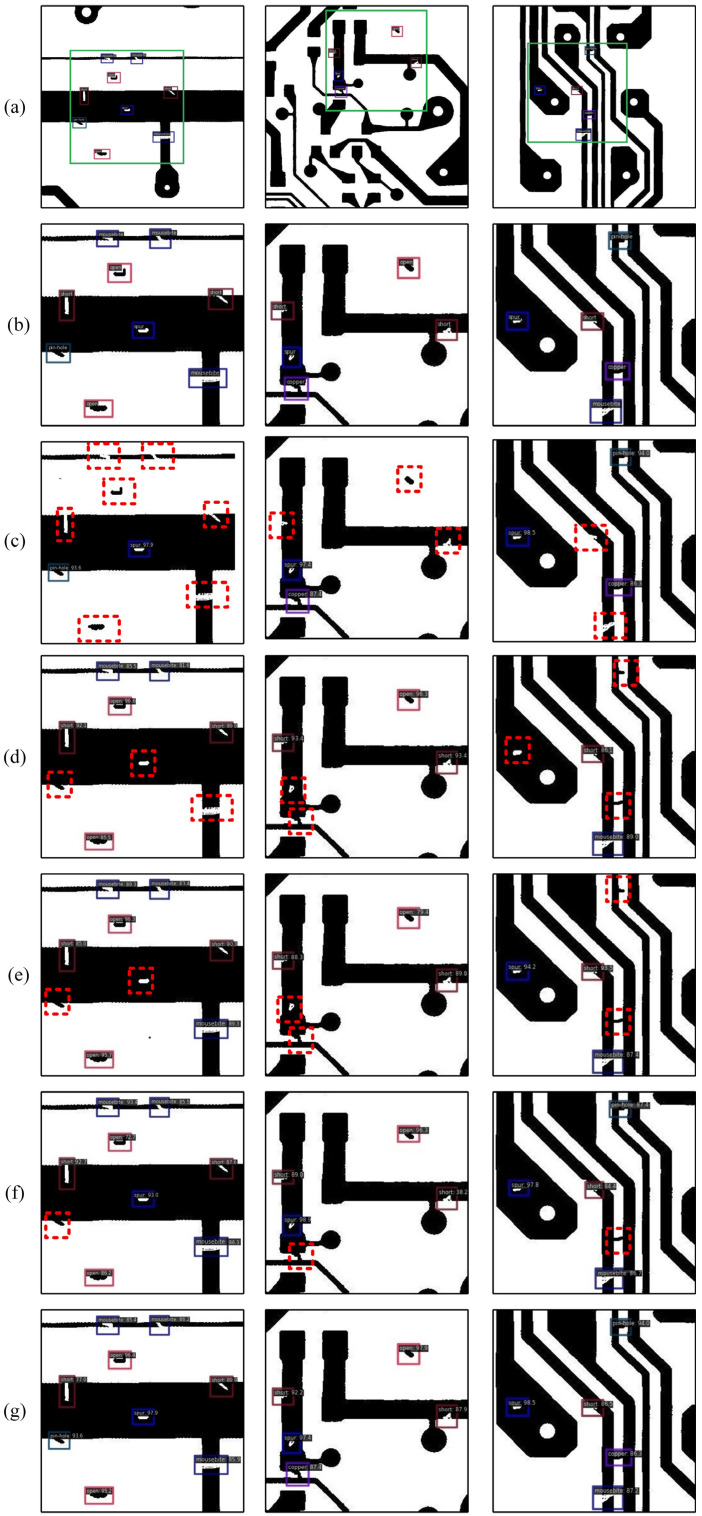
Partial detection results on the DeepPCB dataset. (**a**) Original GT; (**b**) Zoomed-in GT; (**c**) Catastrophic forgetting; (**d**) FSCE; (**e**) IKLF; (**f**) ERD; (**g**) SD-IDD (ours).

Figure 7 presents partial detection results of SD-IDD, Catastrophic forgetting, FSCE, IKLF, and ERD on the DeepPCB dataset. Given that the defect targets in DeepPCB are extremely small, we utilized green rectangular boxes in the original images to highlight defect locations, accompanied by localized zoom-in views. Each column corresponds to the detection results of the same image. The first row displays the original Ground Truth images. The second row shows the zoomed-in Ground Truth images. The third row shows the zoomed-in detection results of catastrophic forgetting caused by the fine-tuning method. The fourth, fifth, sixth, and seventh rows show the zoomed-in detection results of FSCE, IKLF, ERD, and the proposed SD-IDD, respectively. As illustrated in Figure 7, the fine-tuning method forgot 7, 3, and 2 old-class defects, respectively; FSCE missed 3, 2, and 3 defects, respectively; IKLF missed 2, 2, and 2 defects, respectively; ERD missed 1, 1, and 1 defect, respectively; the proposed SD-IDD method perfectly detected all 9, 5, and 5 defects, respectively. Overall, the proposed SD-IDD demonstrated superior small object detection capability on the DeepPCB dataset, making it better suited to tackle the challenges of real-world PCB defect detection tasks.

## 5. Discussion

### 5.1. Effectiveness Discussion

The effectiveness of the SD-IDD method stems from the design of its three distillation strategies. Initially, the high-confidence classification distillation strategy filters out key discriminative information from semantic confusion regions between old and new classes, thereby mitigating the noise interference caused by global classification distillation. This enables the student model to maintain the category boundaries of old classes while learning new ones, effectively alleviating catastrophic forgetting. In the ablation study, this strategy showed an increase of 3.7% and 0.6% in mAP_old compared to global classification distillation on the NEU-DET and DeepPCB datasets, demonstrating its advantage in preserving discriminative knowledge of old classes.

Next, the dual-stage cascaded regression distillation strategy combines GFLv1’s probabilistic distribution regression mechanism with KL divergence distillation, better retaining the uncertainty information in the teacher model’s predictions. Through dual-stage filtering, it ensures the effective transfer of key localization knowledge. The IoU-weighted mechanism enhances the gradient contribution of critical targets, significantly improving the student model’s localization capability. In industrial defect detection, where targets are often densely distributed with fuzzy boundaries, such as the inclusion defects in NEU-DET, SD-IDD achieves higher regression accuracy than other methods, with its predicted boxes perfectly overlapping the GT Box.

Finally, the IoU-driven difficulty-aware feature distillation strategy proposes a dynamic resource allocation mechanism. Due to the variations in shape, scale, and boundary complexity among defect targets, their learning challenges vary greatly. If global feature distillation is employed, the model tends to focus excessively on easy-to-learn targets, while overlooking more difficult ones. In this research, IoU was utilized to quantify regression difficulty, and a dynamic threshold mechanism was implemented to bias distillation resources toward harder targets. This allows the student model to focus more on difficult feature regions during training. This strategy not only preserves the stability of the old class feature space but also enhances the learning efficiency of new class features, achieving a balance between stability and plasticity.

From the perspective of practical industrial deployment, compared with standard single-stage detectors, SD-IDD inevitably introduces additional computational overhead and GPU memory consumption during the training phase due to the distillation strategies at the classification, regression, and feature levels. However, unlike incremental methods that rely on sample replay, SD-IDD does not employ additional data caching or historical sample storage mechanisms. Therefore, it does not incur extra long-term memory usage and data management costs, which provides advantages in better maintainability, data security, and compliance in industrial environments. It is worth emphasizing that the above additional overhead is mainly concentrated in the training phase. During inference, SD-IDD maintains the same network structure as GFLv1 without adding extra branches or inference modules. Thus, it does not compromise detection speed or real-time performance, which is beneficial for deployment in industrial inspection scenarios with high real-time requirements.

### 5.2. Limitations

Table 3, Table 4, Table 5, Table 6, Table 7, Table 8 and Table 9 reveal that the performance of SD-IDD on the DeepPCB dataset is significantly better than that on the NEU-DET dataset. This disparity primarily arises from the differences in defect morphology and background characteristics between the two datasets. The defects in DeepPCB display regular geometric shapes, clear boundaries, sparse and independent distributions, and clean background regions with no texture patterns similar to defects. These attributes provide more explicit discriminative cues for SD-IDD, enabling both classification and localization to achieve high accuracy. Conversely, the defects in NEU-DET present complex and diverse shapes, blurred boundaries, dense adhesive distributions, and backgrounds that share textural similarities with the defects. These factors considerably increase the difficulty for SD-IDD in distinguishing foreground from background and discriminating between various defect categories, resulting in decreased detection accuracy.

SD-IDD still has its own methodological limitations. First, the detection performance of the method highly depends on the prediction quality of the teacher model, and the student model can hardly surpass the teacher model in detection accuracy on old classes. When the teacher model produces errors for a certain class, the student model will inherit and even amplify such errors. Second, the effectiveness of all three distillation strategies relies on the settings of Intersection over Union (IoU) and confidence thresholds. Although this study adopts an adaptive mechanism for thresholds based on the mean and variance of samples, the key scaling factors (e.g., α,β) still require manual tuning. In scenarios with extremely imbalanced data distributions or highly irregular defect shapes, the robustness of threshold settings still needs to be improved. Third, this research does not integrate sample replay or generative memory mechanisms and only relies on distillation to preserve knowledge of old classes. In long-sequence incremental learning tasks, the cumulative effect of knowledge forgetting is still unavoidable, especially for classes learned in the early stages, which gradually degrade as the number of distillation steps increases.

In summary, SD-IDD effectively alleviates catastrophic forgetting through selective distillation mechanisms at the classification, regression, and feature levels. Nonetheless, further improvements are necessary concerning teacher model dependency, threshold robustness, dataset complexity, and long-term incremental object detection. Future work will attempt to integrate sample replay, generative adversarial networks, or more efficient distillation scheduling strategies to further improve the stability and generalization ability of the model.

## 6. Conclusions

This research addressed the problem of incremental defect detection in industrial quality inspection scenarios. We propose a novel method called SD-IDD to tackle issues such as noise interference, low localization accuracy, and feature space rigidity present in existing knowledge distillation-based methods. Targeted distillation strategies are designed at three levels: classification, regression, and feature levels. SD-IDD adopts a high-confidence classification distillation strategy and distills only high-confidence anchors at the classification head, thereby retaining old class knowledge in semantic confusion regions and effectively mitigating catastrophic forgetting. Furthermore, SD-IDD employs a dual-stage cascaded regression distillation strategy, which filters out low-IoU noisy anchors through geometric prior coarse filtering and statistical fine filtering. This strategy improves localization accuracy and forms a positive feedback loop of “filter-distill-accuracy enhancement”. In addition, SD-IDD introduces an IoU-driven difficulty-aware feature distillation strategy, which allocates more distillation resources to hard targets, achieving a balance between the feature learning abilities of old and new classes.

Experimental results on the NEU-DET and DeepPCB datasets indicated that SD-IDD significantly mitigated catastrophic forgetting without using old class samples, achieving mAP_all of 63.6% and 98.3%, which represents a 20.9% and 35.6% improvement compared to fine-tuning methods, respectively. Furthermore, SD-IDD outperforms advanced methods such as ERD, IKLF, and FSCE in overall performance, while ensuring real-time detection. SD-IDD not only confirms the effectiveness and synergy of the three proposed distillation strategies in incremental defect detection but also presents a viable technical solution to address dynamic changes in defect classes in industrial quality inspection.

## Figures and Tables

**Table 1:** Meanings of the symbols in Equation (1).

Symbols	Meaning
$C_{T}$	The output of the teacher model’s classification head
$C_{SO}$	The output of the old classes in the student model’s classification head
$B_{T}$	The output of the teacher model’s regression head
$B_{S}$	The output of the student model’s regression head
$F_{T}$	The output of the teacher model’s FPN feature layers
$F_{S}$	The output of the student model’s FPN feature layers

**Table 2:** Class division of datasets.

Dataset	Initial Phase	Incremental Phase
NEU-DET	crazing	pitted_surface
	inclusion	rolled-in_scale
	patches	scratches
DeepPCB	open	spur
	short	copper
	mousebite	pin-hole

**Table 3:** Ablation study on NEU-DET.

	Classification Head			Regression Head			FPN			mAP_old	mAP_new	mAP_all
	None	CR	ALL	None	BR	ALL	None	FR	ALL			
1	√			√			√			17.3%	68.1%	42.7%
2	√				√			√		51.6%	62.2%	56.9%
3			√		√			√		54.5%	67.9%	61.2%
4		√		√				√		54.1%	61.3%	57.7%
5		√				√		√		53.6%	69%	61.3%
6		√			√		√			54.8%	62.6%	58.7%
7		√			√				√	56.1%	67.7%	61.9%
8		√			√			√		**58.2%**	**69%**	**63.6%**

**Table 4:** Ablation study on DeepPCB.

	Classification Head			Regression Head			FPN			mAP_old	mAP_new	mAP_all
	None	CR	ALL	None	BR	ALL	None	FR	ALL			
1	√			√			√			30.5%	94.9%	62.7%
2	√				√			√		72.2%	97.2%	84.7%
3			√		√			√		98.7%	92.7%	95.7%
4		√		√				√		76.5%	97.3%	86.9%
5		√				√		√		98.7%	93.3%	96.0%
6		√			√		√			77%	97.2%	87.1%
7		√			√				√	99.1%	93.7%	96.4%
8		√			√			√		**99.3%**	**97.3%**	**98.3%**

**Table 5:** Varying α and β on NEU-DET.

α	β	mAP_old	mAP_new	mAP_all
1	1	58.2%	68.6%	63.4%
1	2	57.6%	68.8%	63.2%
2	1	57.6%	69%	63.3%
2	2	58.2%	69%	63.6%

**Table 6:** Varying γ on NEU-DET.

γ	mAP_old	mAP_new	mAP_all
0.5	57.8%	68.4%	63.1%
0.8	57.7%	68.1%	62.9%
adapting	58.2%	69%	63.6%

**Table 7:** Varying Top-K on NEU-DET.

Top-K	mAP_old	mAP_new	mAP_all
80	57.7%	68.3%	63.0%
90	57.8%	68.6%	63.2%
100	58.2%	69%	63.6%
120	58%	68.4%	63.2%

**Table 8:** Comparative experiments on NEU-DET.

Methods	mAP_old	mAP_new	mAP_all
Joint training (Upper Bound)	69.8%	76%	72.9%
Catastrophic forgetting	17.3%	68.1%	42.7%
FSCE [43]	62.3%	38.5%	50.4%
IKLF [44]	**64.7%**	50.3%	57.5%
ERD [40]	57.2%	62.2%	59.7%
SD-IDD (ours)	58.2%	**69%**	**63.6%**

**Table 9:** Comparative experiments on DeepPCB.

Methods	mAP_old	mAP_new	mAP_all
Joint training (Upper Bound)	99.3%	98.9%	99.1%
Catastrophic forgetting	30.5%	94.9%	62.7%
FSCE [43]	69.1%	63.8%	66.4%
IKLF [44]	96.3%	89.3%	92.8%
ERD [40]	97.8%	95.2%	96.5%
SD-IDD(ours)	**99.3%**	**97.3%**	**98.3%**

## Data Availability

The datasets generated or analyzed during this research are available from the corresponding author on reasonable request.
